# Longitudinal Association between Sport Participation and Depressive Symptoms after a Two-Year Follow-Up in Mid-Adolescence

**DOI:** 10.3390/ijerph17207469

**Published:** 2020-10-14

**Authors:** Diego Gómez-Baya, Luis Calmeiro, Tânia Gaspar, Adilson Marques, Nuno Loureiro, Miguel Peralta, Ramón Mendoza, Margarida Gaspar de Matos

**Affiliations:** 1 Department of Social, Developmental and Educational Psychology, Universidad de Huelva, 21071 Huelva, Spain; ramon@uhu.es; 2 School of Applied Sciences, Abertay University, Dundee DD1 1HG, UK; l.calmeiro@abertay.ac.uk; 3 School of Medicine/ISAMB Environmental Health Center, 1000-001 Lisbon, Portugal; tania.gaspar@lis.ulusiada.pt (T.G.); amarques@fmh.ulisboa.pt (A.M.); nloureiro@ipbeja.pt (N.L.); miguel.peralta14@gmail.com (M.P.); mmatos@fmh.ulisboa.pt (M.G.d.M.); 4 School of Psychology, Universidade Lusiada, 1000-001 Lisbon, Portugal; 5 Projeto Aventura Social, 1000-001 Lisbon, Portugal; 6 CIPER, Faculdade de Motricidade Humana, Universidade de Lisboa, 1000-001 Lisbon, Portugal; 7 School of Education, Instituto Politécnico de Beja, 7800 Beja, Portugal; 8 Faculdade de Motricidade Humana, Universidade de Lisboa, 1000-001 Lisbon, Portugal

**Keywords:** adolescents, depression, health promotion, physical activity, mental health, wellness

## Abstract

Sport participation has been advocated as a strategy to improve adolescents’ mental health. However, how these variables evolve during the adolescent years is uncertain. The objective of the present work was to examine the longitudinal associations of sports participation and depressive symptoms in adolescents. Participants were 525 Spanish adolescents (50.3% boys), aged between 12 and 15 years old (M = 13.45, SD = 0.69). They were enrolled in 18 secondary schools located in Andalusia (Spain) in the first two academic years of Compulsory Secondary Education. Data were collected in three waves, separated by one year, by administering self-report measures of depressive symptoms and sport participation. Repeated measures analyses of variance were conducted to examine the change in depressive symptoms and sport participation, and a latent growth curve model was tested to examine the change in depressive symptoms controlling initial sport participation and gender. At each wave, more frequent sport participation was related to lower depressive symptoms. Two years after the first assessment, adolescents became more depressed but not more active. Less active adolescents had more depressive symptoms in each assessment time. At all moments of assessment, girls were less active and reported more depressive symptoms. Moreover, girls had a higher increase in depressive symptoms than boys. Promotion of physical activity (PA) as a mental health strategy needs to consider gender differences as boys and girls have different patterns of presentation of depressive symptoms throughout adolescence.

## 1. Introduction

Adolescence is characterized by considerable biological and psychological changes that render individuals more prone to psychosocial stress and impaired functioning [1]. The onset of depression is commonly observed in adolescence; its incidence upsurges dramatically after puberty with yearly prevalence rates higher than 4%. In fact, the cumulative probability of depression has been shown to increase from 5% in early adolescence to 20% at end of this period [2]. This rise in depression is more significant in girls than in boys [2,3]. Because depression significantly impacts young people’s quality of life (e.g., somatization, truancy and poor academic performance, poor interpersonal relationships, engagement in risk behaviors, eating disorders, suicidal ideation), health promotion professionals should emphasize the development of protective factors. Physical activity (PA) may be one of such factors [4,5]. Thus, more research is needed to examine the role of PA in depressive symptoms by gender in adolescent samples.

The associations between PA and mental health are well documented [6]. Although its efficacy in clinical samples is debatable [7], the data concerning non-clinical samples are more consensual [8,9]. Overall, reviews and studies suggest that lower levels of PA are associated with higher levels of depression in adolescents, but the evidence is limited because most studies are cross-sectional [4,10,11,12]. In the absence of well-designed randomized controlled trials, longitudinal studies provide an opportunity for researchers to explore the direction of the relationship between PA and mental health.

The direction of the relationship between PA and depression remains uncertain in adolescence. Some studies do not support a longitudinal relationship, whether PA is measured through self-report [13] or objective measures [14,15]. In a 10-year cohort study, Birkland et al. [13] observed an inverse relationship between PA and depression in adolescents. However, neither baseline levels of PA predicted later changes in depression nor baseline levels of depression predicted later changes in PA. Olive et al. [16] also failed to find that baseline levels of depression at age 8 were associated with PA at age 16. However, a significant inverse relationship with physical fitness was observed in girls. Furthermore, in a 3-year cohort study, Toseeb et al. [15] found that objectively measured PA in 15-year-olds did not predict depressive symptoms in later adolescence, suggesting that the effect of PA may be small or inexistent during adolescence. These studies seem to indicate that depression does not operate as a barrier for PA or that PA does not protect against the development of depression in adolescents.

Other studies do support the existence of a longitudinal association between PA and depression. For example, Raudsepp and Vink [17] conducted a 2-year longitudinal investigation with female adolescents and observed a unidirectional relationship, so that baseline levels of depressive symptoms predicted changes in PA and sleep disturbances; however, PA did not predict later depression. Notwithstanding, in a 6-year study, Jerstad et al. [18] found a bidirectional relationship between PA and depression in female adolescents, so that participants who were more active at baseline had fewer depressive episodes, while participants who had more depressive symptoms at baseline were less physically active. All these relationships were modest at best, but the effect of depression on PA was more robust than the inverse effect. While PA may constitute a protective factor for the onset of depression, the anhedonia and motor retardation symptoms typical of depression are likely to limit involvement in PA [18].

Kandola et al. [19] further studied the longitudinal associations between depression and PA by using clinical interviews and accelerometers to measure these variables. It was found that light PA, moderate-to-vigorous PA (MVPA), and total PA were inversely associated with depressive symptoms at age 18. Between the ages of 12 and 16, for each daily hour of additional light PA, depression scores decreased 8% to 11%. Additionally, a 15-min increase in MVPA at age 12 was associated with a 9% decrease in depression at age 18. Based on accelerometer data as well, Langguth, Schmid, Gawrilow, and Stadler [20] studied individual same-evening and next morning changes in depressed mood following PA in girls. A 60-min increase in MVPA over the individual’s mean predicted a 50% decrease in depression in the next morning. No significant effects were found for boys. It is possible that engagement in less structured and more cooperative activities, developmental dissimilarities in self-esteem, and body image or motives for participation contribute to these gender differences.

Straatman et al. [21] also observed gender differences in longitudinal associations between MVPA, sedentary behavior, and depression in a large sample of Brazilian boys and girls. While higher levels of baseline depression symptoms predicted lower levels of PA at a 2-year follow-up in boys, poor baseline wellbeing was associated with increased screen time in girls. These results suggest the need for differentiated health promotion targets for boys and girls. No longitudinal association between objectively measured PA and the development of depressive symptoms was observed in this large population-based sample. These results do not support the hypothesis that PA protects against developing depressive symptoms in adolescence.

Sport participation is an important context for young people’s development. While the sport may be associated with unhealthy behaviors such as interpersonal violence and the use of alcohol [22] and drug use [23], as well as injuries, support for the beneficial impact on wellbeing, social development, skills development, or academic achievement has been found [23,24]. Concerning its effects on depression, Doré et al. [25] found evidence that both recreational and performance sport participation during childhood predicted mental health in adolescence, regardless of gender. Zarrett et al. [24] found that sport participation significantly predicted lower depression with better results when participation was concurrent with other community activities (e.g., religion). In a cross-sequential longitudinal study of three cohorts, Wang, Chow, and Amemiya [26] predicted lower depressive symptomatology as a result of participation in team sports, but not in individual sports. Similarly, in a five-year longitudinal study, Sabiston et al. [27] demonstrated that depressive symptoms in early adulthood could be predicted by participation in team sport during adolescence. It is thought that team sports are beneficial for developing interpersonal relationships, social support, and a sense of belonging, main developmental tasks for adolescents [27].

Given that the longitudinal relationship between PA and depression is still unclear, more investigations focusing on providing further insight are important. The rising challenges of depression during adolescence and in the developmental transition to adulthood and the need to develop evidence-based efficient mental health-promoting strategies justify this study. As well, the study of possible gender differences in sport participation and its effect on depressive symptoms in adolescence is also necessary. Thus, its aim is to analyze the prospective association between sport participation and depressive symptoms during mid-adolescence, by examining possible gender differences. In line with previous research, we hypothesize a decrease in sport participation and an increase in depressive symptoms. Girls are expected to report more depressive symptoms and less sport participation. Moreover, a negative prospective relationship is expected between sport participation and depressive symptoms.

## 2. Materials and Methods

### 2.1. Participants and Study Design

A longitudinal design with three assessment timepoints was employed in this study. Participants were 525 Spanish adolescents (50.3% boys), with ages ranging from 12 to 15 years (M = 13.45, SD = 0.69). They were enrolled in 18 secondary schools located in Andalusia (Spain) in the first two academic years of Compulsory Secondary Education. A heterogeneous sample was collected, by selecting, by convenience, public (*n* = 5) and private secondary schools (*n* = 13) from the eight Andalusian provinces representing rural (*n* = 4), semi-urban (*n* = 4), and urban contexts (*n* = 10). In each secondary school, the respective participating classrooms were randomly selected. At baseline, 878 adolescents completed the self-report measures. One year later, approximately 76% participated in the second assessment (*n* = 670). In the final assessment, 525 adolescents completed the measures. Hence, 145 adolescents abandoned the study, thus reaching attrition of 21.6%. This overall attrition (39.8%) may be due to a change of educational center or lack of attendance in the day of the fieldwork. Concerning ethnicity, most participants were Caucasian (99.2%) and 0.8% were Latin so that Spanish was the primary language.

### 2.2. Instruments

Depressive symptoms. The Spanish version of the Brief Children’s Depression Inventory was used [28,29]. In previous research, this 10-item inventory presented satisfactory psychometric properties for assessing both the presence and severity of depressive symptoms in children and adolescents and it was as valid for research as the longer version of this instrument [29]. Each item measures a different depressive symptom, and three possible response options are presented (0 = “no symptoms”, 1 = “mild severity”; 2 = “maximum severity”). A total score is obtained by adding all items and it ranges from 0 to 20, with a higher score indicating a greater presence of depressive symptomatology. In the present study, this instrument showed good internal consistency (α = 0.73 at time 1, α = 0.74 at time 2, and α = 0.76 at time 3).

Sport participation. A single indicator to assess extracurricular sport participation was used. Participants answered the question ‘How often do you practice sports or gymnastics during the out-of-school time?’ Five Likert-type response options were presented: never (1), rarely (2), one day a week (3), several days a week but not every day (4), and every day (5). This question was validated and used in the international Health Behavior in School-aged Children study [30,31].

### 2.3. Data Collection Procedure

A 2-year longitudinal study was conducted with three assessments separated by one year each. In each assessment, participants individually completed a paper-and-pencil self-report survey during their classroom time. The survey was administered by a member of the research team. No student refused to participate in the study and the omission of answers was below 0.5%. Participants did not obtain any reward for their participation. To track the participants and maintain anonymity during follow-up assessments, a numeric code was attributed to each participant. All principles embodied in the Declaration of Helsinki were respected, and the adolescents voluntarily participated. Written informed consent was obtained from all parents and adolescents, and the approval from the University of Huelva’s ethics board was previously received (project AP2009-4621).

### 2.4. Data Analysis

Descriptive statistics (i.e., mean and standard deviation) were calculated for depressive symptoms and sport participation in the three waves. Gender and age differences were analyzed using Student t-tests, calculating Cohen’s d for size effect. Repeated measures analyses of variance were conducted to examine the change in depressive symptoms and sport participation, as a function of gender and age. To examine the change in depressive symptoms, we controlled for baseline sport participation and gender. Partial correlations were performed controlling for gender and age, considering low correlation with r around 0.1, medium, around 0.3, and large, greater than 0.5. Little test indicated that missing values were completely at random, χ^2^(33) = 40.54, *p* = 0.172. All analyses were conducted with SPSS 21.0, using an α level of 0.05 for all statistical tests.

A latent growth curve model was tested with the program EQS 6.1, which allows the estimation of the trajectory of change of variables as well as the effect of other variables on those changes [32]. The Model fit was based on covariance matrices using the Maximum Likelihood Estimation method [33]. A linear latent growth curve model was developed for the change in depressive symptoms based on scores in Time 1, Time 2, and Time 3. The intercept represents the initial values of depressive symptoms in each wave, and the slope factor describes the change over time. Effects of sport participation at the beginning of the study and demographics were examined in both intercept and slope. Chi square, Confirmatory Fit Index (CFI), and root mean square error of approximation (RMSEA) were examined as overall fit indexes [34] and standardized solutions were reported.

## 3. Results

### 3.1. Descriptive Statistics and Gender Differences

Table 1 presents the descriptive statistics for depressive symptoms and sport participation in the three waves, as well as the analysis of gender differences. Low means were found in depressive symptoms and moderate scores were observed in sport participation. Girls reported more depressive symptoms than boys in each assessment time, with greater differences at the end of the study. Concerning sport participation, boys showed higher means than girls, with greater differences also at the end of the study.

### 3.2. Analysis of Change and Bivariate Correlations

With regards to change in the study variables across time, results indicated an increase in depressive symptoms, F(2, 464) = 4.75, *p* = 0.009, ηp^2^ = 0.02. This change was significant among girls, F(2, 243) = 7.17, *p* = 0.001, ηp^2^ = 0.06, but not among boys, F(2, 219) = 0.26, *p* = 0.769. No significant change was observed in frequency of sport participation for the whole sample, F(2, 518) = 0.49, *p* = 0.615; neither for boys, F(2, 257) = 0.70, *p* = 0.499, nor girls, F(2, 259) = 0.23, *p* = 0.792, sport participations changed significantly. School of enrollment did not interact with the changes in depressive symptoms, F(17, 448) = 1.19, *p* = 0.269 or in sport participation, F(17, 502) = 1.12, *p* = 0.331.

Partial correlations between depressive symptoms and sport participation, controlling for gender and age, are described in Table 2. There were large positive associations between the assessments of depressive symptoms in each timepoint, as well as between the assessments of sport participation. Small associations were observed between measures of sport participation and depressive symptoms. Sport participation in time 1 was negatively related to depressive symptoms in time 1. The same result was found with measures in time 2. Moreover, depressive symptoms in time 1 were also negatively associated with sport participation in timepoints 2 and 3. No significant association was detected between sport participation and depression in time 3.

### 3.3. Change in Depressive Symptoms by Initial Sport Participation and Gender

**Figure 1:**
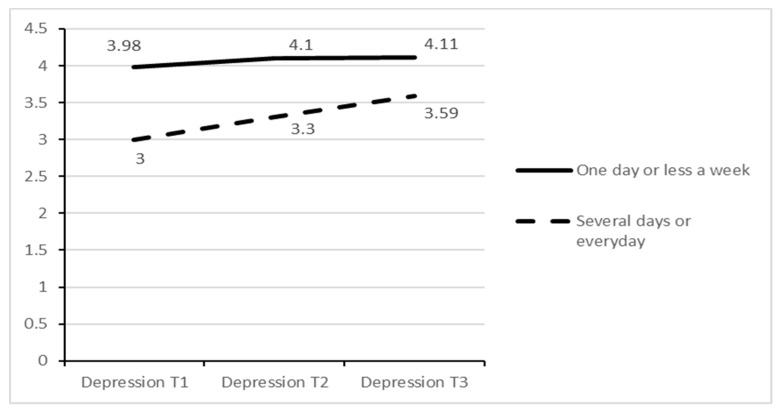
Changes in depressive symptoms by frequency of sport participation.

Results showed that change in depressive symptoms interacted with sport participation at time 1, F(8, 456) = 2.04, *p* = 0.039, ηp^2^ = 0.02, and with gender, F(2, 456) = 4.09, *p* = 0.017, ηp^2^ = 0.01. However, interaction between gender x sport participation had no effect, F(8, 456) = 0.49, *p* = 0.863. Thus, a greater increase in depressive symptoms was observed in girls and in participants with high initial sport participation, who began the study with lower scores in depression (Figure 1).

**Figure 2:**
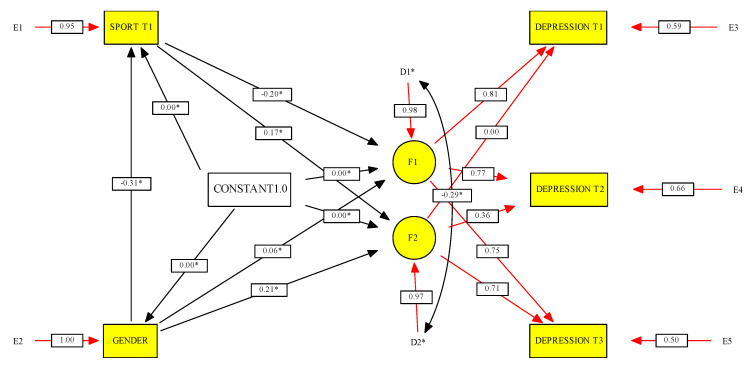
Latent growth curve model. * Significant at *p* < 0.05.

Figure 2 presents the latent growth curve model of the change in depressive symptoms from time 1 to time 3, taking into consideration the effects of gender and sport participation at time 1. The model reached good overall fit, χ^2^(3, *n* = 525) = 0.54, *p* = 0.911, CFI = 1.00, RMSEA = 0.01, 90% confidence interval RMSEA = 0.00–0.06. Results indicate that: (a) the intercept (F1) and slope (F2) of depressive symptoms were negatively associated, so a greater increase in depression was observed among participants with lower initial levels; (b) gender had a significant effect on the slope of depressive symptoms, so a greater increase was found in girls, but there was no effect on the intercept; (c) sport participation in Time 1 presented a negative effect on depressive symptoms’ intercept, so adolescents who practiced more sport at wave one reported less depressive symptoms in subsequent waves; (d) sport participation in Time 1 showed a positive effect on depressive symptoms’ slope, so a greater increase in depressive symptoms was detected among participants with more frequent sport participation at the beginning of the study; (e) gender had a negative effect on sport participation at time 1, so boys reported more frequent practice than girls. Concerning explained variance, both the intercept and the slope reached a value of R^2^ = 0.05. Standardized solutions are presented in Figure 2.

## 4. Discussion

The aim of this study was to examine the prospective relationship between sport participation and depressive symptoms during mid-adolescence. In the present study, adolescents’ depressive symptoms and sport participation were assessed at the age of 13–14 and followed for two years, totaling three assessment moments. Consistent with our hypothesis, findings showed that more frequent sport participation was related to fewer depressive symptoms. These results corroborate those found by North et al.’s study [35] with a community sample of adolescents and by Craft and Landers’s study [36] with adolescents aged 12 to 18 years old, who demonstrated that exercise significantly reduced depression among participants with clinical depression and depression resulting from mental illness. One recent study has reported that adolescents who engage in higher frequencies of PA are more resilient to developing depressive symptoms [37]. Moreover, as hypothesized, throughout the present study, period adolescents became more depressed. These results are consistent with an increased prevalence of depression during the adolescent years [2]. Less active adolescents had more depressive symptoms in each assessment time. However, more active adolescents at baseline presented a bigger increase over time in depressive symptoms. In line with our hypothesis, regardless of assessment moment, girls reported less frequent sport participation and more depressive symptoms than boys. Furthermore, the increase in depressive symptoms through time was higher among girls than among boys. This evidence has been reported by McMahon et al. [38].

The present results are consistent with previous studies that suggest that PA has a positive effect on reducing depressive symptoms, which supports the recommendation of PA as a strategy to promote mental health in youth [6,10,19]. However, the debate about the causality of the relationship and its gender specificity is ongoing [6]. On the one hand, it is possible that sports and PA only decrease depressive symptoms when other conditions are met, such as enjoying sports, having friends or a family culture of practice, or being competent. Additionally, beneficial effects can depend on personal characteristics, as the ability to take pleasure from practice, socialize, and relax or be rewarded by practice. On the other hand, sport participation may sometimes emphasize competitiveness or the development of physical fitness rather than the promotion of health and wellbeing. Therefore, sport participation is often found to be associated with violence, alcohol consumption, and injuries [22,23]. Nevertheless, Doré et al. [25] found that both competitive and recreational sport participation predicted lower levels of depression in young adulthood with stronger effects being observed for adolescents who have accumulated more years of participation. Continued participation may represent the fulfillment of basic psychological needs; hence, beneficial effects can be fostered if basic adolescents’ needs can be met, such as autonomy, competence, and connection.

Regarding gender, results are mixed and often suggest that adolescents, especially girls, tend to abandon sport participation due to lack of time, dissatisfaction with sport practice, and insufficient variety of suitable sport activities, or for other reasons [39]. Therefore, adolescents should be given a voice so that policymakers and practitioners may understand what diverts them away from PA and sports, especially girls. The practice of PA decreases as young people grow up when they become more autonomous and responsible for their choices. More research is needed to explore why parents when their children were younger oblige them to practice PA regardless of their tastes and interests. Moreover, in many cases, parents are not a direct model for promoting physical exercise, implicitly sending the message, for example, with expressions such as “if you are grown-up you do not need to do physical exercise” or “if I want to be grown-up, I do not exercise”. Furthermore, other important influence in adolescence is the group of friends, which could not value the practice of PA and the young person could not have the socio-emotional skills to resist peer pressure. The young person may feel a lot of pressure from parents and teachers to study and to succeed at school and may not have time enough to practice PA, or maybe young people invest their energy in romantic relationships and sexual relationships. Furthermore, some of the young people who continue to engage in PA also are those who reveal more self-regulation, assertiveness, goal setting, and pursuit skills, and those skills could explain the lowest depressive symptoms.

Girls practice less PA and experience higher depressive symptoms. Regardless of studying the relationship between these variables, they are verified independently of each other. More research is needed to understand why girls practice less PA, to address the social, educational, and parental reasons. Normally, when a healthy behavior is not frequently practiced by a sector of the population, it could be explained in terms of the barriers that are found by that population group when considering that practice. Gender differences in PA practice during adolescence that are found in many European countries suggest that girls find more barriers than boys for being physically active. The nature of these barriers should be explored in further research in order to make it easier for girls choosing and practicing an active lifestyle. Girls tend to internalize their sadness/distress, their feelings, increasing depressive symptoms, and decreasing interest and energy for other activities, namely PA. Boys who tend to externalize their sadness/distress, often through intense PA, express and manage this sadness/distress.

PA and sports are good for mental health, but a stronger calling for the promotion of socio-emotional skills is needed, namely the development of intentional self-regulation that may help adolescents thrive. Young people need to develop skills, find alternatives, capacity for reflection, decision-making, and inherent accountability that bring autonomy and well-being, even better if associated with a healthy and pleasurable PA. Increasing activity levels, exercise intensity, and sports participation among the least active young people should be a target of community and school-based interventions to promote well-being. The influence of sport participation on positive youth development is greater when it is concomitant with engagement in other community activities [24].

The present study presents some limitations. First, to measure sport participation a single item was used, which focus on one dimension of participation, namely frequency. Therefore, studies should differentiate between types, intensity, and duration of sport activities. Second, the type of sport was not taken into consideration. Adolescents who participated in team sports have been found to report higher levels of happiness than those who played individual sports [40]. Therefore, it will be useful to analyze the type of sport (e.g., cooperative vs. competitive, individual vs. group, structured vs. unstructured, aerobic vs. anaerobic) as a moderating variable of the relationship between sport participation and depression. Third, the measure of depression was based on self-report. Although the measure used is valid and reliable, a stronger approach would be to use diagnostic interviews to reduce the self-report bias of depressive symptoms, which is not always feasible in large studies. Finally, to argue for causation, randomized controlled trials are needed to study short and long-term effects of sport participation on depression [41].

## 5. Conclusions

In summary, this manuscript provides longitudinal evidence for the prospective negative association between sport participation and depressive symptoms in mid-adolescence. Both PA and depressive symptoms remain a gendered issue, in the call for educational and clinical attention. Following previous research, girls are less active and present more depressive symptoms. During adolescence, depressive symptoms show a higher increase in girls than in boys.

This study highlights that sports participation may help to promote mental health and well-being in adolescence. This study surfaces an important message for public policies, namely in the area of education, health, and gender equity, looking for ways of promoting girls’ wellbeing, social competences, and self-confidence and helping them to find “a PA that fits each of them” in order to be active, healthy, and happy.

## Figures and Tables

**Table 1:** Descriptive statistics and gender differences.

	Mean (Standard Deviation)			
		Gender Differences		
Variables	Total	Boys	Girls	*t*-Tests
1. Depressive symptoms Time 1	3.25 (2.57)	2.92 (2.64)	3.59 (2.46)	*t*(519) = −2.97, *p* = 0.003, d = 0.26
2. Depressive symptoms Time 2	3.57 (2.71)	3.15 (2.59)	3.96 (2.77)	*t*(419) = −3.33, *p* = 0.001, d = 0.30
3. Depressive symptoms Time 3	3.72 (2.81)	3.09 (2.71)	4.32 (2.78)	*t*(493) = −4.96, *p* < 0.001, d = 0.45
4. Frequency of Sport Participation Time 1	3.41 (1.28)	3.80 (1.19)	3.02 (1.25)	*t*(523) = 7.34, *p* < 0.001, d = 0.64
5. Frequency of Sport Participation Time 2	3.38 (1.23)	3.78 (1.13)	2.97 (1.19)	*t*(519) = 7.99, *p* < 0.001, d = 0.70
6. Frequency of Sport Participation Time 3	3.43 (1.26)	3.86 (1.09)	2.99 (1.28)	*t*(522) = 8.40, *p* < 0.001, d = 0.73

**Table 2:** Partial correlations between depressive symptoms and frequency of sport participation in all waves, controlling for gender and age.

Variables	1	2	3	4	5	6
1. Depressive symptoms Time 1	1					
2. Depressive symptoms Time 2	0.54 ***	1				
3. Depressive symptoms Time 3	0.43 ***	0.59 ***	1			
4. Sport Participation Time 1	−0.16 **	−0.08	−0.03	1		
5. Sport Participation Time 2	−0.14 **	−0.11 *	−0.05	0.65 ***	1	
6. Sport Participation Time 3	−0.10 *	−0.09	−0.07	0.51 ***	0.59 ***	1

Note. * *p* < 0.05; ** *p* < 0.01; *** *p* < 0.001.
